# Cationic Gas-Permeable Mold Fabrication Using Sol–Gel Polymerization for Nano-Injection Molding

**DOI:** 10.3390/gels10070453

**Published:** 2024-07-11

**Authors:** Sayaka Miura, Rio Yamagishi, Mano Ando, Arisa Teramae, Yuna Hachikubo, Yoshiyuki Yokoyama, Satoshi Takei

**Affiliations:** 1 Department of Pharmaceutical Engineering, Toyama Prefectural University, Imizu 939-0398, Toyama, Japan; sayaka13579@outlook.jp (S.M.); rio.yamagishi@outlook.jp (R.Y.); mano_ando@outlook.jp (M.A.); u118020@st.pu-toyama.ac.jp (A.T.); y.hachikubo@outlook.jp (Y.H.); 2 Toyama Industrial Technology Research and Development Center, Takaoka 933-0981, Toyama, Japan; yokoyama@itc.pref.toyama.jp

**Keywords:** sol–gel polymerization, cationic gas-permeable mold, injection molding, nano-fabrication, hydrophobic materials, nano-structure

## Abstract

Cationic gas-permeable molds fabricated via sol–gel polymerization undergo cationic polymerization using epoxide, resulting in gas permeability owing to their cross-linked structures. By applying this cationic gas-permeable mold to nano-injection molding, which is used for the mass production of resins, nano-protrusion structures with a height of approximately 300 nm and a pitch of approximately 400 nm were produced. The molding defects caused by gas entrapment in the air and cavities when using conventional gas-impermeable metal molds were improved, and the cationic gas-permeable mold could be continuously fabricated for 3000 shots under non-vacuum conditions. The results of the mechanical evaluations showed improved thermal stability and Martens hardness, which is expected to lead to the advanced production of resin nano-structures. Furthermore, the surface roughness of the nano-protrusion structures fabricated using injection molding improved the water contact angle by approximately 46°, contributing to the development of various hydrophobic materials in the future.

## 1. Introduction

Rapid technological progress requires the development of new materials, nano-structures, and multi-component composites with specific chemical and physical properties to meet modern technological requirements [1]. Sol–gel polymerization is a synthetic method used for designing advanced catalytic formulations based on metals and metal oxides with a high degree of structural and compositional homogeneity [2]. The pioneering discovery of sol–gel polymerization was attributed to Ebelmen, who first reported it in 1846 while researching the production of silica glass [3]. The hydrolysis and condensation of inorganic precursors to form siloxane bonds lead to the formation of sols, and over time, a reaction occurs in which these colloidal particles aggregate to form a porous, three-dimensional network, or gel [4]. Sol–gel polymerization has the advantages of low processing temperatures [5,6,7], high homogeneity [8,9], and the ability to produce materials in a variety of forms, such as coatings, thin films, and powders [10,11].

Sol–gel polymerization is very widely used [12,13,14] and a wide range of application developments have been investigated [15]. The methods have been applied to optical materials [16], catalytic materials [17,18], medical materials [19], electronics [20], energy-related materials [21], and insulation materials [22]. For example, cost-effective and environmentally friendly gas sensors fabricated using sol–gel polymerization have the potential to reduce the negative impact of climate change in the future by detecting volatile organic compounds (VOCs) and other toxic gases [23]. Crespo-Monteiro et al. also developed a sol–gel polymerization that allows the direct micro-nano structuring of ZrO_2_ layers without an etching process, using optical or nano-imprint lithography [24]. In addition, as a medical material, Catauro et al. reported silica/polyethylene glycol (PEG) hybrid materials containing chlorogenic acid (CGA) synthesized via the sol–gel polymerization, providing clear evidence of their anti-proliferative activity against cancer cells [25].

In particular, TiO_2_-based materials are of great interest for a wide range of applications including photocatalysis, catalysis, sensing, and energy conversion [26]. For example, Julien et al. reviewed the environmentally friendly production process of TiO_2_ photocatalysts using a colloidal aqueous sol–gel polymerization, which yields crystalline materials without calcination [27]. Thus, high-performance materials prepared using the sol–gel polymerization are increasingly being studied and worldwide [28].

We developed the TiO_2_-SiO_2_ cationic gas-permeable mold using attractive sol–gel polymerization. The ultraviolet (UV) irradiation of the cationic sol–gel material obtained via sol–gel polymerization leads to the cationic polymerization of the epoxide, forming a cross-linked structure. The cross-linked structure provides the material with gas permeability.

Bubble formation in nano-imprint lithography is a common problem that causes molding defects in the process [29,30,31]. To address this issue, research institutes have developed methods to prevent gas entrapment via nano-imprinting under vacuum or reduced pressure conditions [32] or in CO_2_ or helium environments [33] to reduce molding defects. However, neither method can be applied without increasing manufacturing complexity and cost [34]. Other methods include nano-imprint lithography with PDMS molds utilizing their high gas permeability, which eliminates air bubbles and allows the transfer of microstructures [35]. In our previous studies, the use of gas-permeable molds improved defects during the nano-imprint process [36,37].

**Figure 1:**
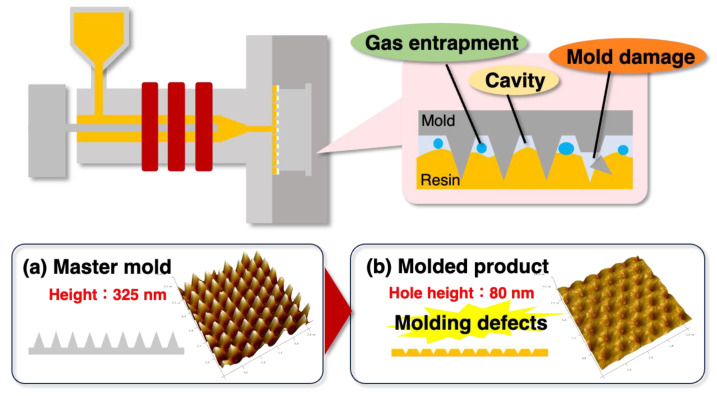
Molding defects when performing nano-injection molding using conventional gas-impermeable molds.

The convex master mold shown in Figure 1a was inserted into an injection molding machine, and injection molding was performed in a non-vacuum environment. As with nano-imprinting, when a conventional gas-impermeable mold such as a metal is used, the mold is insufficiently filled and gas in the air is trapped, resulting in a molding defect in the concave molded product, as shown in Figure 1b, owing to insufficient transfer. 

In this study, cationic gas-permeable molds of TiO_2_-SiO_2_ prepared using sol–gel polymerization were fabricated, and their mechanical evaluation results showed that the thermal stability and Martens hardness were improved. Cationic gas-permeable molds have been successfully applied not only to nano-imprinting but also to injection molding, which is commonly used for the mass production of resins [38] and to improve molding defects such as those caused by gas entrapment. Under non-vacuum conditions, the same cationic gas-permeable mold was used to fabricate PP nano-protrusion structures with a height of 300 nm, which could be nano-injection-molded continuously for 3000 shots. When the contact angle with water was measured using the fabricated nano-protrusion structures, the contact angle was improved by approximately 46° compared with that of the flat PP films. The nano-injection molding method using the cationic gas-permeable mold fabricated using the sol–gel polymerization in this study may contribute to the advanced production of resin nano-structures and the development of specific materials for a variety of applications.

## 2. Results and Discussion

### 2.1. Dynamic Viscoelasticity Measurement Results

**Figure 2:**
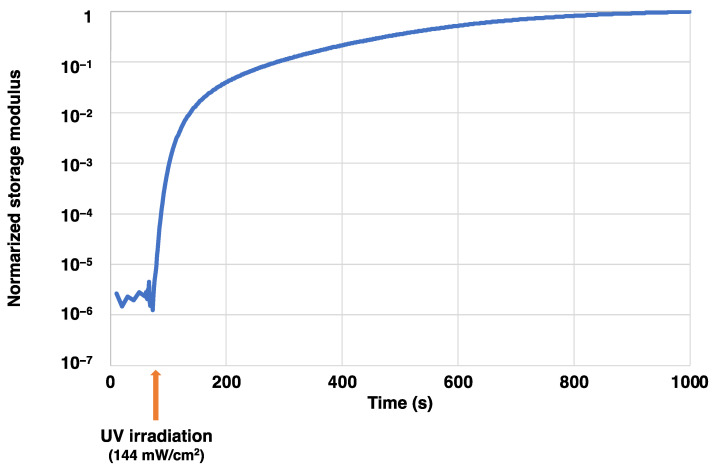
Dynamic viscoelasticity measurements of cationic sol–gel material.

Figure 2 shows the dynamic viscoelasticity measurement results of the cationic sol–gel material. The horizontal axis represents time and the vertical axis represents the normalized storage modulus, standardized to 1 at 1000 s. The UV intensity was 144 mW/cm^2^. The curve of the cationic sol–gel material changed rapidly after UV irradiation (60 s after the start of the test). From Figure 2, it was considered that the cationic sol–gel material based on TiO_2_-SiO_2_ was 78% cured at 140 s (20.2 J/cm^2^) and 88% cured at 340 s (49.0 J/cm^2^) after UV irradiation. Cationic polymerization using epoxide occurred in the presence of cationic initiators, and the storage modulus increased.

### 2.2. Fourier Transform Infrared Spectroscopy (FT-IR) Spectra Changes Due to UV Irradiation

**Figure 3:**
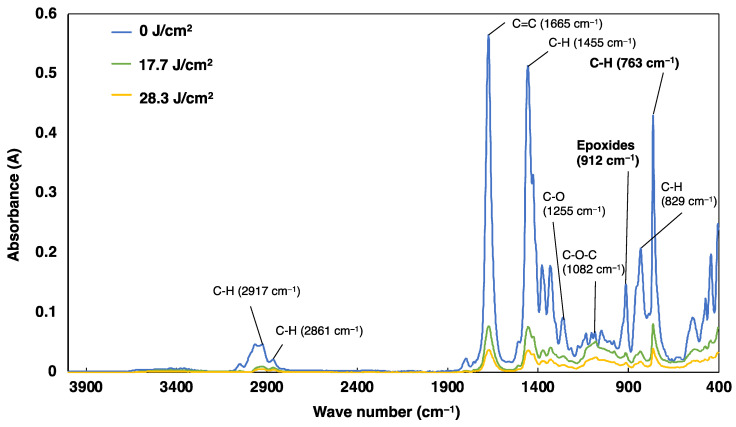
UV-doses dependence of the cationic sol–gel materials on FT-IR spectra.

Figure 3 shows the FT-IR spectra of the cationic sol–gel materials at different doses of UV irradiation (0, 17.7, and 28.3 mJ/cm^2^). The spectra of the cationic sol–gel materials showed the C-H stretching vibration of alkanes (2917 cm^−1^, 2861 cm^−1^), the C=C stretching vibration of benzene rings (1665 cm^−1^), the C-H bending vibration of alkanes (1455 cm^−1^), the C-O- of esters (1255 cm^−1^), the C-O-C of ethers (1082 cm^−1^), epoxides (912 cm^−1^), the C-H out-of-plane vibration of benzene rings (829 cm^−1^), and five neighboring H of benzene rings (763 cm^−1^).

The target spectrum for comparing cationic polymerization using epoxide in the cationic sol–gel materials was approximately 910 cm^−1^. We also compared the decrease in epoxide at 762 cm^−1^ in the spectral region (770–735 cm^−1^) of the five neighboring hydrogen atoms of the benzene rings of cationic initiators, whose structures were not expected to change before and after UV irradiation. Peak intensity ratios were calculated by placing a base point at each end of each peak and quantifying the height between the baseline and spectra. The peak intensity ratios were 0.292 before UV irradiation, 0.128 at 17.7 J/cm^2^, and 0.126 at 28.3 J/cm^2^ after UV irradiation.

The cationic sol–gel material showed a decrease in epoxide content at a UV irradiation of 17.7 J/cm^2^. An additional UV irradiation of 28.3 J/cm^2^ did not result in significant changes in the epoxide. Rings of epoxy cyclohexane in sol–gel copolymers and cross-linking agents have been shown to exhibit higher polymerization rates than other types of epoxide functional groups, such as alkyl glycidyl ethers, and the rate may decrease after the rapid reaction [39].

These results confirmed the cationic polymerization of the cationic sol–gel materials using FT-IR spectroscopy.

### 2.3. Mechanical Strength Measurement Results

**Figure 4:**
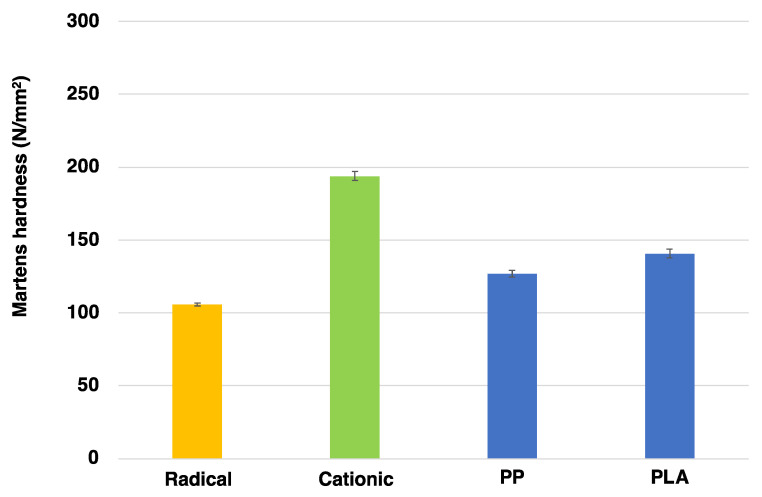
Mechanical strength (Martens hardness) of radical gas-permeable mold, cationic gas-permeable mold, PP, and PLA.

Figure 4 shows the results of mechanical strength measurements using the nano-indenter. The Martens hardness of our previous studies of the radical gas-permeable mold [40,41] and the cationic gas-permeable mold were 106 and 194 N/mm^2^, respectively. These results indicate that the cationic gas-permeable mold has higher strength (approximately 1.8 times) than the radical gas-permeable mold, and improved durability can be expected with respect to filling pressure and other factors when injection molding is performed. In addition, polypropylene (PP), which is often used as a general-purpose plastic [42], and polylactic acid (PLA), which has attracted much attention as a bio-based biodegradable plastic [43], with Martens hardnesses of 127 and 141 N/mm^2^, respectively, showed relatively low mechanical strength. In contrast to the general problem of mold damage in injection molding, it was suggested that when PP and PLA were injection-molded using a cationic gas-permeable mold, the possibility of mold damage was relatively low, and the same mold could be used repeatedly as the mold had higher mechanical strength than the applicable resin.

### 2.4. Thermogravimetry Analysis (TGA) Results

**Figure 5:**
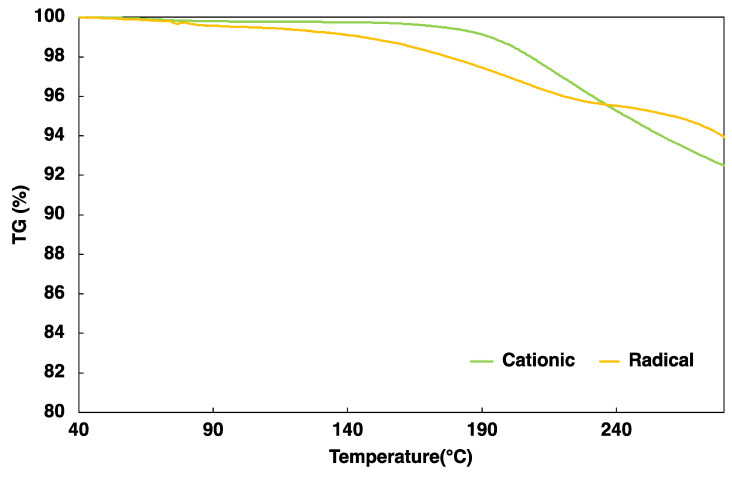
Thermogravimetric analysis of radical gas-permeable mold, cationic gas-permeable mold.

Figure 5 shows the TG curves for the cationic gas-permeable mold at temperatures ranging from 40 to 280 °C. The TG curves show the mass loss of the cationic gas-permeable mold; similarly, the radical gas-permeable molds from the previous studies [40,41] were also measured and compared.

It was observed that the cationic gas-permeable mold was stable with little weight loss from 40 to 160 °C, with thermal decomposition occurring mainly at approximately 165 °C. Furthermore, the thermal decomposition of the radical gas-permeable mold occurred gradually, mainly at 80 °C, indicating that it was less heat-resistant than the cationic gas-permeable mold.

The percentage of weight loss was 1.4% for the cationic gas-permeable mold and 3.1% for the radical gas-permeable mold at 200 °C. The cationic gas-permeable mold is considered to exhibit good heat resistance owing to the presence of epoxide [44,45]. As can be seen from these results, the cationic gas-permeable mold has better thermal resistance than the radical gas-permeable mold and can be used for heat molding below 160 °C.

### 2.5. Nano-Injection Molding Results

**Figure 6:**
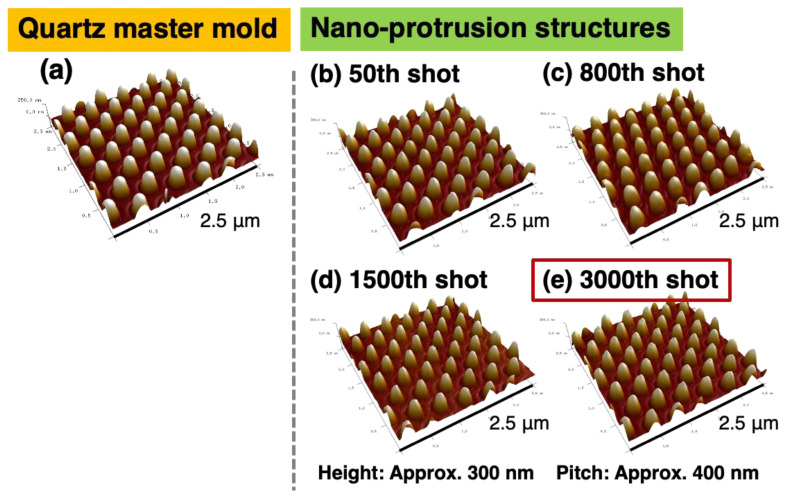
SPM images of mold surface and nano-protrusion structures: (**a**) quartz master mold, (**b**) 50th shot, (**c**) 800th shot, (**d**) 1500th shot, and (**e**) 3000th shot of nano-protrusion structures.

Figure 6 shows scanning probe microscopy (SPM) images of PP-derived nano-protrusion structures obtained via nano-injection molding. Figure 6a shows the quartz master mold, Figure 6b shows the 50th shot, Figure 6c shows the 800th shot, Figure 6d shows the 1500th shot, and Figure 6e shows the 3000th shot of the PP nano-protrusion structures.

When a cationic gas-permeable mold was fabricated by using the quartz master mold and nano-injection molding was performed using the cationic gas-permeable mold, nano-protrusion structures could be fabricated without molding defects [40] owing to gas entrapment during nano-injection molding, which has been difficult in the past. After 3000 shots of continuous molding using the same mold, there was almost no difference in the pattern between 50 and 3000 shots, improving the shots of successful patterning in nano-injection molding by 7.5 times compared with the results of 400 shots of continuous molding achieved in the previous studies [40]. Furthermore, a comparison of the bottom diameters from the SPM images of the quartz master mold and the 3000th shot showed that the former had an average diameter of 248 nm, whereas the latter had an average diameter of 239 nm (±4.9 nm).

Compared with the conventional radical gas-permeable mold, the successful results of 3000 shots on the general-purpose injection molding machine using the developed sol–gel-derived cationic gas-permeable mold are expected to be mass-producible and low-cost.

### 2.6. Water Contact Angle Measurement Results

**Figure 7:**
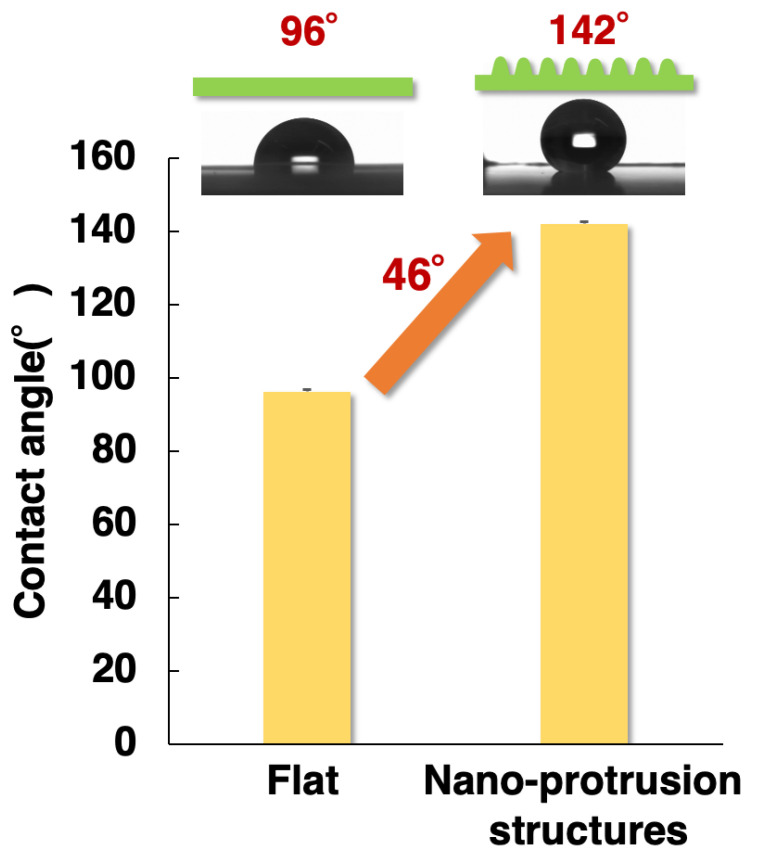
Comparison of water contact angles on flat surface and surface with nano-protrusion structures.

Figure 7 shows the comparison of the water contact angle measurements on a flat surface before patterning and the obtained nano-protrusion structures after patterning. The average water contact angle of the flat PP without nano-protrusion structures was approximately 96° (±8°). In contrast, the nano-protrusion structures had an average water contact angle of approximately 142° (±6°), with 147° being the best. In the same PP, the water contact angle improved by 46° when the surface was given nano-protrusion structures.

In a previous study [46], micrometer line patterns in a high-fluorine-containing UV-curable resin increased the water contact angle by approximately 20° compared with a flat. In this study, the contact angle was significantly improved by the addition of protruding nano-structures compared with the line patterns, in agreement with the discussion of Kaga et al. [47].

Surface wettability is important in a variety of practical applications, such as in colloid science and chemistry in everyday life [48]. Wettability control can be achieved on surfaces with micro- or nano-scale surface roughness [49], and there is currently research into the different hydrophobic behaviors of nano-structures depending on their size and shape [50]. In the future, further reduction in the nano-structure size and advanced nano-injection molding will contribute to the development of practical applications in a wide range of fields, such as superhydrophobic materials.

## 3. Conclusions

Cationic gas-permeable mold was prepared via the UV irradiation of cationic sol–gel material prepared using the sol–gel polymerization, resulting in cationic polymerization. In addition, various evaluations of the cationic gas-permeable mold were performed. First, the change in the FT-IR spectra with respect to the amount of UV irradiation showed a decrease in the epoxide peak (910 cm^−1^), confirming that cationic polymerization had occurred. Next, when the mechanical strength was measured using a nano-indenter, it had a Martens hardness 1.8 times higher than that of the radical gas-permeable mold in our previous study and was even higher than that of PP and PLA. In addition, the thermogravimetric analysis showed that there was almost no weight loss from 40 to 160 °C, which was stable, and thermal decomposition was observed at 165 °C. In addition, when the temperature was increased to 200 °C, the weight loss was approximately 1.4%. The prepared cationic gas-permeable mold was used for nano-injection molding. When using conventional gas-impermeable metal molds, there are problems with molding defects due to gas entrapment in the air or cavities. However, by using the cationic gas-permeable mold in this study, we were able to fabricate PP-derived nano-protrusion structures with a height of approximately 300 nm and a pitch of approximately 400 nm without any outstanding defects under non-vacuum conditions. In addition, it was possible to perform 3000 shots of nano-injection molding, which was 7.5 times better than the 400 shots results obtained with the previous radical gas-permeable mold. This is thought to be due to the improved thermal stability and Martens hardness of the cationic gas-permeable mold. Increasing the number of moldings is expected to lead to mass production and lower costs. Water contact angle measurements of the fabricated PP-derived nano-protrusion structures showed an improvement of 46° compared with that of a flat PP surface. This study, which allows the insertion of a cationic gas-permeable mold into a general-purpose injection molding machine and the mass production of nano-protrusion structures, is promising for the development of super-water-repellent materials based on the surface roughness of nano-protrusion structures by realizing nano-injection molding.

## 4. Materials and Methods

### 4.1. Preparation of Cationic Gas-Permeable Mold

**Figure 8:**
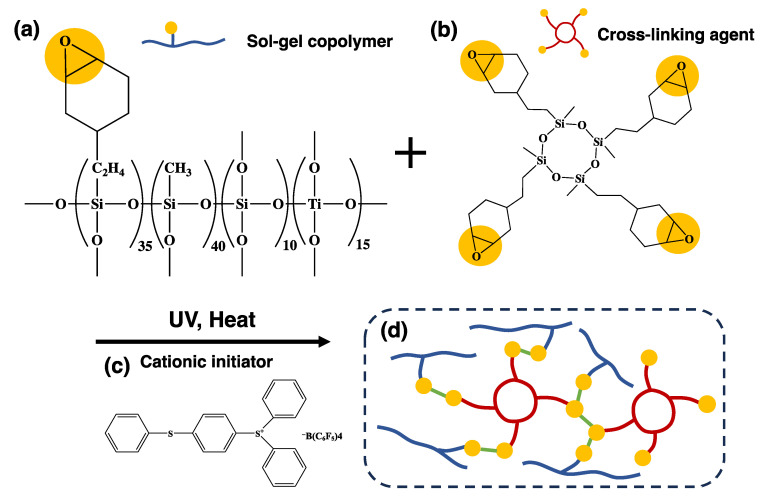
Chemical structures and reaction diagram: (**a**) sol–gel copolymer, (**b**) cross-linking agent, (**c**) cationic initiator, (**d**) schematic diagram of the reaction using cationic polymerization.

To prepare the cationic gas-permeable mold, the sol–gel copolymer of Figure 8a, the cross-linking agent of Figure 8b, and the cationic initiator of Figure 8c were mixed to prepare a cationic sol–gel material. The sol–gel copolymer in Figure 8a (schematic diagram) was synthesized using the conditions of sol–gel polymerization as follows: 35 wt% 2-(3,4-epoxycyclohexyl) ethyltrimethoxysilane (Gelest, Morrisville, NC, USA), 40 wt% methyltrimethoxysilane (Gelest, Morrisville, NC, USA), 15 wt% tetraethyltitanate (Gelest, Morrisville, NC, USA), and 10 wt% tetraethoxysilane (Gelest, Morrisville, NC, USA). The four components were placed in a flask under nitrogen atmosphere and stirred using a magnetic stirrer for 1 h to dissolve them to obtain a mixed solution. The resulting mixed solution was heated to 55 °C and reacted with ion-exchanged water, *p*-toluenesulfonic acid (Tokyo Chemical Industry, Tokyo, Japan), and hydrochloric acid (FUJIFILM Wako Pure Chemical, Osaka, Japan) for 2 h. The reaction solution was then cooled to room temperature, and anhydrous magnesium sulfate (Fujifilm Wako Pure Chemical, Osaka, Japan) was added in appropriate quantities, dehydrated, and filtered. An appropriate amount of activated carbon (Tokyo Chemical Industry, Tokyo, Japan) was added to the filtrate, which was then filtered in the same manner. Excess toluene and ion-exchanged water were removed under reduced pressure to obtain the sol–gel copolymer. The solid content of the sol–gel copolymer was calculated from the weight before and after heating to remove the solvent.

To the 87 wt% sol–gel copolymer, tetrakis [(epoxycyclohexyl) ethyl] tetramethylcyclotetrasiloxane (Gelest, Morrisville, USA) (Figure 8b) was added as a cross-linking agent. Subsequently, 3 wt% 2-hydroxy-2-methyl-1-phenylpropanone (CPI-100B(40), San-Apro, Kyoto, Japan) (Figure 8c) was added to the sol–gel copolymer and cross-linking agent as a cationic initiator.

To confirm the polymerization of the prepared cationic sol–gel material in UV irradiation, the solvent resistance of the resulting cationic sol–gel material was evaluated as follows. The solution of the cationic sol–gel material was applied to a silicon wafer using the casting method, irradiated with a UV spot light source (Lightning cure LC8, Hamamatsu Photonics, Shizuoka, Japan) for 2 min, and then sintered on a hot plate at 120 °C for 10 min. The coating was immersed in toluene for 1 min. The film thickness was measured again and the difference between the initial and final thickness was calculated as the amount of stripping. The stripping test results of less than 10 nm were considered acceptable. The film thickness change was 6.5 nm, indicating good solvent resistance.

Figure 8d shows a schematic diagram of the reaction via cationic polymerization. Gas permeability is ensured by the gaps created by the long chains of the sol–gel copolymer and ring structure of the cross-linking agent.

### 4.2. Dynamic Viscoelasticity Measurements

Dynamic viscoelasticity measurements were performed using a dynamic viscoelasticity measuring device (MCR102, Anton Paar, Graz, Austria), which allows UV irradiation during measurements. The storage modulus of the cationic sol–gel material was measured in oscillatory rotation mode (parallel plate diameter; 12 mm, gap; 0.100 mm, frequency; 3.0 Hz, strain; 0.2%, temperature; 26.1 °C). During measurements, UV irradiation was performed using a UV spot light source (Hamamatsu Photonics, Shizuoka, Japan). The intensity of the UV light was measured using a UV radiometer (ACCU-CALTM-50, DYMAX, Torrington, CT, USA) and was 144 mW/cm^2^. After an interval of 0–60 s without UV irradiation, UV irradiation was started and measurements were performed for 1000 s (approximately 34 min).

### 4.3. FT-IR Measurements

The structural changes in the cationic sol–gel materials owing to the UV cross-linking reaction were examined using FT-IR before and after UV irradiation. The measurements were conducted using an FT-IR system (Spectrum Two, Perkin Elmer, Waltham, MA, USA). Data were recorded at a resolution of 4 cm^−1^, with 10 integrations and a frequency range of 400–4000 cm^−1^. Measurements were performed on materials exposed to a metal halide lamp (59 mW/cm^2^) (DGM2301A-01, Sun Energy, Osaka, Japan) for 0, 5, or 8 min.

### 4.4. Mechanical Strength Measurements

The Martens hardness of the radical gas-permeable mold, the cationic gas-permeable mold, PP, and PLA were measured using a microhardness tester (Fischerscope HM2000, Fischer Instruments, Saitama, Japan); the indentation test was performed according to ISO 14577-1 [51]. The Martens hardness was derived from the load curves obtained during the nano-indentation test, which was performed thrice under a load of 10 mN.

### 4.5. Thermogravimetric Analysis

The radical gas-permeable mold and the cationic gas-permeable mold were UV-cured at the appropriate curing times and the samples were scraped into aluminum cups. The measurements were performed using a thermogravimetric differential thermal analyzer (TG/DTA320, Seiko Instruments, Chiba, Japan) in the temperature range of 40–280 °C (temperature gradient; 10 °C/min). The weights of the analyzed samples ranged from 1.5 to 2.4 mg.

### 4.6. Fabrication of Cationic Gas-Permeable Mold

**Figure 9:**
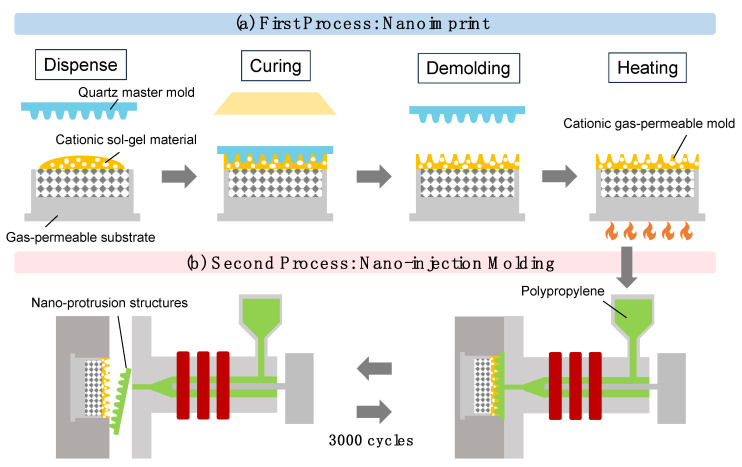
Process diagram: (**a**) nano-imprint processes of the cationic gas-permeable mold, (**b**) nano-injection molding processes of nano-protrusion structures.

Figure 9a shows the nano-fabrication of the above cationic gas-permeable mold via nano-imprint. The gas-permeable substrate of the cationic gas-permeable mold was fabricated using a metal photoengraving composite processing machine (LUMEX Avance-25, Matsuura Machinery Corporation, Fukui, Japan) [52,53]. The laser processing chamber was filled with nitrogen gas to prevent oxidation during the melting of the material. Standard maraging steel powders (average particle size of 20–30 µm) were burn-hardened via irradiation with a 400 W Yb fiber laser; the shapes were cut and this sequence of operations was repeated. The moderate space created by the adhesion between the maraging steel powders contributed to the gas permeability of the gas-permeable substrate. The gas-permeable substrates were ultrasonically cleaned in an ultrasonic cleaner (US-101, SND, Nagano, Japan) using acetone for 20 min. They were then vacuum dried in a vacuum dryer (AVO-250SB, AS ONE, Osaka, Japan) at 180 °C for 20 min to remove lubricants and other oils used in the metal photoengraving composite processing.

The cationic sol–gel material was placed on a gas-permeable substrate prepared using metal photoengraving composite processing. The release from the cationic sol–gel material was improved by releasing the quartz master mold with a release agent (DURASURF DS-831TH, Harves, Saitama, Japan). The quartz master mold was placed on top of the cationic sol–gel material and polymerized using the UV irradiation (17.7 J/cm^2^) of the cationic sol–gel material. The quartz master mold was then released and further polymerized via heat treatment at 60 °C for 5 min [54]. The UV curing process has many advantages, such as fast processing, low energy consumption, and environmentally friendly properties, making it a popular alternative to thermal curing [55].

The nano-injection molding process is shown in Figure 9b. A cationic gas-permeable mold was inserted into the injection molding machine (GL150, Sodick, Kanagawa, Japan), equipped with a screw pre-plaster system, and 3000 injection molding shots were performed. The basic parameters of the injection molding conditions were filling speed (18.5 mm/s), melt temperature (220 °C), molding temperature (30 °C), holding pressure (20 MPa), holding pressure time (10 s) and cooling time (10 s). The resin used was polypropylene (NOVATEC-PP BC03B, Japan Polypropylene, Tokyo, Japan).

### 4.7. SPM Measurement

The shape observation of the quartz master mold and the nano-protrusion structures were performed using a Bruker Dimension Icon system (Bruker, Billerica, MA, USA). The measurement mode was set to PeakForce Tapping AFM mode, and the observation was performed using a Silicon AFM probe OMCL-AC240TS (Olympus, Tokyo, Japan). The conditions for the quartz master mold were scan size: 2.5 × 2.5 µm^2^; scan rate: 1 Hz; and resolution: 256 × 256 pixels, and for the nano-protrusion structures, scan size: 2.5 × 2.5 µm^2^; scan rate: 0.5 Hz; and resolution: 128 × 128 pixels.

### 4.8. Water Contact Angle Measurement

The water contact angle was measured on PP film without nano-protrusion structures and on PP film with nano-protrusion structures. The water contact angle was measured using a fully automatic contact angle meter (Drop Master DM500, Kyowa Surfaces Science, Saitama, Japan) using the θ/2 analysis method. The drop volume was 1.0 µL and the measurement started immediately after the drop. The measurements were performed at 25 °C. After measuring five different points on the surface, the first and tenth measurements were omitted, and the average value of the water contact angle was calculated. The water contact angle was measured in the direction horizontal to that of the linear structure.

## Data Availability

The datasets generated and/or analyzed during the current study are not publicly available because they belong to ongoing research but are available from the corresponding author upon reasonable request.
